# Differential gene expression in *Anopheles stephensi* following infection with drug-resistant *Plasmodium yoelii*

**DOI:** 10.1186/s13071-017-2326-y

**Published:** 2017-08-29

**Authors:** Jingru Zhang, Jiacheng Huang, Feng Zhu, Jian Zhang

**Affiliations:** 1 Department of Pathobiology, the Third Military Medical University, Chongqing, People’s Republic of China; 2 Students brigade 5, The Third Military Medical University, Chongqing, People’s Republic of China

**Keywords:** Differential gene expression, *Anopheles*, Drug-resistant *Plasmodium*

## Abstract

**Background:**

The transmission of drug-resistant parasites by the mosquito may be influenced by the altered biological fitness of drug-resistant parasites and different immune reactions or metabolic change in the mosquito. At this point, little is known about the variations in mosquito immunity and metabolism when mosquitoes are infected with drug-resistant parasites. To understand the differential gene expression in *Anopheles* following infection with drug-resistant *Plasmodium*, we conducted a genome-wide transcriptomic profiling analysis of *Anopheles stephensi* following feeding on mice with drug-resistant or drug-sensitive *P. yoelii*, observed changes in gene expression profiles and identified transcripts affected by the drug-resistant parasite.

**Results:**

To study the impact of drug-resistant *Plasmodium* infections on *An. stephensi* gene transcription, we analyzed the three major transition stages of *Plasmodium* infections: at 24 h and 13 and 19 days after blood-feeding. Six cDNA libraries (R-As24h, R-As13d, R-As19d,S-As24h, S-As13dand S-As19d) were constructed, and RNA sequencing was subsequently performed. In total, approximately 50.1 million raw reads, 47.9 million clean reads and 7.18G clean bases were obtained. Following differentially expressed gene (DEG) analysis, GO enrichment analysis of DEGs, and functional classification by KEGG, we showed that the variations in gene expression in *An. stephensi* infected by the drug-resistant *P. yoelii* NSM occurred mainly at 13 days after blood meal during sporozoite migration through the hemolymph. The differentially expressed genes included those functioning in some important immune reaction and iron metabolism pathways, such as pattern recognition receptors, regulators of the JNK pathway, components of the phagosome pathway, regulators of the melanization response, activators of complement reactions, insulin signaling cascade members, oxidative stress and detoxification proteins.

**Conclusions:**

Our study shows that drug-resistant *P. yoelii* NSM has an impact on the transcript abundance levels of *An.stephensi* mostly at 13 days after blood meal during sporozoite migration through the hemolymph and that most differentially expressed genes were downregulated. Our results highlight the need for a better understanding of selective pressures from these differentially expressed genes of the drug-resistant *Plasmodium* in the mosquito and the different transmission patterns of drug-resistant *Plasmodium* by *Anopheles*.

**Electronic supplementary material:**

The online version of this article (doi:10.1186/s13071-017-2326-y) contains supplementary material, which is available to authorized users.

## Background

The emergence and spread of drug-resistant parasites continue to be major problems in relation to malaria control and elimination [1]. The spread of drug resistance may depend on the transmission of drug-resistant parasites by the mosquito. Some studies have shown an association between the spread of drug resistance and levels of transmission of drug-resistant parasites by the mosquito. For example, the capacity of artemisinin-resistant parasites to infect such highly diverse *Anopheles* species may explain the rapid expansion of these parasites in Cambodia and neighboring countries and further compromises efforts to prevent their global spread [2]. In contrast, parasites resistant to the antimalarial atovaquone fail to transmit by mosquitoes, and the lack of transmission suggests that resistance will be unable to spread in the field, greatly enhancing the utility of atovaquone in malaria control [3]. The possible reasons for the transmission of drug-resistant parasites by the mosquito may come from drug-resistant parasites with altered biological fitness [4] and mosquitoes with different effective immune clearance or metabolic changes against drug-resistant parasites. At this point, little is known about the variations in mosquito immunity and metabolism when mosquitoes are infected with drug-resistant parasites. Therefore, to investigate differential gene expression in *Anopheles* following infection with drug-resistant *Plasmodium*, we conducted a genome-wide transcriptomic profiling analysis of *An.stephensi* following feeding on mice infected with drug-resistant or drug-sensitive *P. yoelii*, and we chose the three major transition stages associated with *Plasmodium* infection, including24h after infection with *P. yoelii*, 13 days following sporozoite migration through the hemolymph, and 19 days following sporozoite invasion of the salivary glands.

## Methods

### Mosquito rearing, parasite lineage and infection of mice

*Anopheles stephensi* (Hor strain) mosquitoes were raised at 24 °C, 75% humidity under a 12:12 light-dark photocycle. The mosquitoes were maintained on a 5% sucrose solution during the adult stage. The NSM strain used in this analysis is a mefloquine-resistant clone selected from *P. yoelii* NS [5, 6]. These parasites were obtained from Malaria Research and Reference Reagent Resources [7]. BALB/c mice were randomly divided into two groups (groups 1 and 2). Each mouse of group 1 was inoculated intraperitoneally with 200 μl of infected blood containing approximately 1 × 10^6^ infected red blood cells of NSM (iRBCs), while each mouse of group 2 was infected with the same number of *P. yoelii* NS. The course of infection was tracked by Giemsa-stained blood smear analysis. The smears were prepared from tail blood samples collected at different time points after inoculation. When the gametocytemia reached 1%, female mosquitoes were fed on anesthetized mice for 2 h and collected at 24 h, 13 days and 19 days (d) after blood-feeding. Four-day-old adult females were allowed to blood-feed on animals from one of the following two groups: NSM-infected mice (R, infected with drug-resistant *Plasmodium*) or *P. yoelii* N-infected mice (S, infected with drug-sensitive *Plasmodium*). Unfed mosquitoes were removed from the groups.

### RNA extraction, quantification and integrity determination

Mosquitoes were collected, and each assay was performed with 20 mosquitoes, which were washed in ice-cold 95% ethanol to remove cuticle lipids. Mosquitos were subsequently rinsed in ice-cold water. All carcasses from the same group were combined, frozen in liquid nitrogen, and ground into fine powder for RNA extraction using TRIzol reagent (Life technologies, Carlsbad, CA, USA). RNA purity was checked using a NanoPhotometer® spectrophotometer (IMPLEN, Westlake Village, CA, USA). RNA concentration was measured using a Qubit® RNA Assay Kit and a Qubit® 2.0 Fluorometer (Life Technologies, Frederick, MD, USA).

### Library preparation and transcriptome sequencing

A total of 3 μg of RNA per sample was used as input material for the RNA sample preparations. Sequencing libraries were generated using the NEBNext® Ultra™ RNA Library Prep Kit for Illumina® (NEB, Ipswich, MA, USA) following the manufacturer’s recommendations. Briefly, mRNA was purified from total RNA using poly-T oligo-attached magnetic beads. Fragmentation was performed using divalent cations at an elevated temperature in NEBNext First Strand Synthesis Reaction Buffer (5×). First strand cDNA was synthesized using random hexamer primers and M-MuLV Reverse Transcriptase (RNase H-). Second strand cDNA synthesis was subsequently performed using DNA Polymerase I and RNase H. Remaining overhangs were converted into blunt ends *via* exonuclease/polymerase activities. After adenylation of 3′ ends of DNA fragments, NEB Next Adaptors (with hairpin loop structures) were ligated to the cDNA fragments to prepare for hybridization. Six cDNA libraries (including R-As24h, R-As13d, R-As19d,S-As24h, S-As13d and S-As19d) were constructed, and RNA-seq was performed by a commercial company (Novogene, Beijing, China). To select cDNA fragments that were 150–200 bp in length, the library fragments were purified using the AMPure XP system (Beckman Coulter, Beverly, USA). Next, 3 μl of USER Enzyme (NEB, USA) was incubated with size-selected, adaptor-ligated cDNA at 37 °C for 15 min. This incubation step was followed by a second incubationstep for 5 min at 95 °C prior to PCR. PCR was subsequently performed with Phusion High-Fidelity DNA polymerase, Universal PCR primers and Index (X) Primer. Finally, PCR products were purified (AMPure XP system, Beckman Coulter, Sykesville, MD, USA), and library quality was assessed on the Agilent Bioanalyzer 2100 system. Clustering of the index-coded samples was performed on a cBot Cluster Generation System using a TruSeq PE Cluster Kit v3-cBot-HS (Illumina) according to the manufacturer’s instructions. After cluster generation, the library preparations were sequenced on an Illumina HiSeq platform, and 125-bp/150-bp paired-end reads were generated.

### Sequence read cleanup and mapping to genome

Raw data (raw reads) in fastq format were first processed using in-house Perl scripts. In this step, clean reads were obtained by removing reads containing adapter sequences, reads containing ploy-N sequences and reads with low quality scores (< Q20). At the same time, the percentages of reads with Q20 and Q30 and the GC contents were calculated. All of the downstream analyses were based on clean data with associated quality scores ≥ Q20. The analyzed RNA sequencing data for this study were submitted to the Sequence Read Archive (SRA) at NCBI and can be viewed under the BioProject number PRJNA374479, BioSample number SAMN06323937. Reference genome and gene model annotation files were downloaded directly from the Vectorbase website (https:// www.vectorbase.org/organisms/anopheles-stephensi). The *An.stephensi* genome was assembled using Bowtie v2.2.3 [8, 9], and paired-end clean reads were aligned to this reference genome using TopHat v2.0.12 [10, 11]. TopHat was selected as the mapping tool because it can generate a database of splice junctions based on the gene model annotation file; this approach generates more optimal mapping results compared with other non-splice mapping tools [12].

### Quantification of gene expression levels and differential expression analysis

HTSeq v0.6.1was used to count the number of reads mapped to each gene [13], allowing us to quantify gene expression levels, which were expressed as fragments per kilobase per millions or FPKM [14]. Prior to differential gene expression analysis for each sequenced library, the read counts were adjusted using the edgeR program package through one scaling normalized factor [15]. Differential expression analysis was performed using the DEGSeq R package (1.20.0), and the *P*-values were adjusted using the Benjamini & Hochberg method. A corrected *P*-value of 0.05 and a log2 (fold-change) of 1 were set as the thresholds for significantly differential expression [16, 17].

### Quantitative real-time PCR analysis of selected differentially expressed gene

Quantitative real-time PCR was performed to confirm RNA sequencing expression profiles, and nine differentially expressed genes were selected for validation of the expression data (Additional file 1: Figure S1). Gene-specific primers and SYBR Premix EX Taq (TaKaRa, Japan) were used on an ECOTM Real-Time PCR System (Illumina, USA) during real-time PCR analysis. The reaction mixture (15 μl total volume) contained 7.5 μl of reaction buffer, 0.45 μl of primers, 6.05 μl of ddH_2_O and 1 μl of cDNA template. Ribosomal protein S7 (rpS7) mRNA (GenBank: AF539918) was used as an internal control for data normalization with the forward and reverse primers (Additional file 1: Figure S1).

## Results and discussion

### RNA-seq analysis of differential gene expression in *Anopheles stephensi* following infection with drug-resistant *P. yoelii*

To study the effects of infection with drug-resistant *P. yoelii* on *An. stephensi* gene transcription, six RNA samples were prepared from adult female mosquitoes in the drug-resistant *Plasmodium* infection group (R) and the drug-sensitive *Plasmodium* infection group (S) at 24 h, 13 d and 19 d after blood-feeding. These time points are three major transition stages of *Plasmodium* infections in the mosquito, representing ookinete invasion of the midgut, sporozoite migration through the hemolymph and sporozoite invasion of the salivary glands. During these stages, the parasites experience considerable loss of abundance in the mosquitoes. Following the removal of reads flagged as low quality or low complexity, approximately 50.1 million raw reads, 47.9 million clean reads and 7.18G clean bases were obtained. Among the raw reads, ~97% had Phred quality scores (QS) of 20 or higher, and ~92% of the reads had QS30 or higher, with an estimated error rate of ~0.02%. The GC contents were approximately 49–51% for all of the sequences (Table 1). These results suggest that the sequencing reads generated from the RNA samples exhibited excellent quality.

**Table 1:** Sequence reads, read quality, and error rates

Sample name	Raw reads	Clean reads	Clean bases	Error rate (%)	Q20 (%)	Q30 (%)	GC content (%)
R_As24h	50,921,692	48,696,660	7.3G	0.02	96.79	91.95	50.91
S_As24h	48,149,668	45,974,494	6.9G	0.02	96.62	91.61	50.96
R_As13d	53,198,176	50,759,128	7.61G	0.02	96.49	91.41	49.43
S_As13d	48,863,236	46,720,102	7.01G	0.02	96.57	91.51	50.12
R_As19d	51,480,496	49,154,934	7.37G	0.02	96.59	91.57	50.71
S_As19d	45,488,110	43,544,700	6.53G	0.02	96.69	91.72	50.95

*Note*: Raw reads, sequence reads from each RNA sample; Clean reads, reads following the removal of adapters, low quality reads or reads with 'N' > 1%; Clean bases, read number times read length; Error rate, calculated error rate based on read quality; Q20 (%) and Q30 (%), percentage of bases with *Phred* score > 20 or 30; GC content (%), percentage of G plus C in the sequences

### Mapping sequence reads to chromosomes

The clean reads from each sample were mapped to the assembled chromosomes of the *An.stephensi* genome using TopHat v2.0.12; these sequences are available at the Vectorbase website, and ~70% of the reads were initially assigned to chromosomes. Additional ‘filters’ (mismatch = 2 and read edit distance = 3) were applied to remove low-quality non-specific mapped reads. The new analyses mapped ~53% of the reads to the chromosomes, with split reads (with gaps) encompassing ~17% of the reads. A total of ~0.8% of the reads resulted in multiple hits; these hits may have represented reads from multi-gene families. The mapped reads were distributed evenly across the *An. stephensi* chromosomes. Approximately 79–83% of the mapped reads were located at exons, and ~14–19% were located at intergenic regions or 5′/3′ untranslated regions (UTRs), with relatively even distributions in the ‘+’ and ‘−’ DNA strands (Table 2).

**Table 2:** Statistics of sequence reads mapped to chromosomes

Sample name	R_As24h	S_As24h	R_As13d	S_As13d	R_As19d	S_As19d
Total reads	48,696,660	45,974,494	50,759,128	46,720,102	49,154,934	43,544,700
Total mapped (%)	33,863,898 (69.54)	31,833,061 (69.24)	32,893,865 (64.8)	31,436,501 (67.29)	33,605,516 (68.37)	30,132,506 (69.2)
Multiple mapped (%)	360,551 (0.74)	335,293 (0.73)	390,620 (0.77)	375,385 (0.8)	387,500 (0.79)	354,761 (0.81)
Uniquely mapped (%)	33,503,347 (68.8)	31,497,768 (68.51)	32,503,245 (64.03)	31,061,116 (66.48)	33,218,016 (67.58)	29,777,745 (68.38)
Reads map to ‘+’ (%)	16,759,346 (34.42)	15,759,435 (34.28)	16,246,792 (32.01)	15,544,924 (33.27)	16,616,630 (33.8)	14,899,272 (34.22)
Reads map to ‘−’ (%)	16,744,001 (34.38)	15,738,333 (34.23)	16,256,453 (32.03)	15,516,192 (33.21)	16,601,386 (33.77)	14,878,473 (34.17)
Non-splice reads (%)	25,443,056 (52.25)	23,876,914 (51.94)	24,708,873 (48.68)	22,802,795 (48.81)	24,659,025 (50.17)	22,113,465 (50.78)
Splice reads (%)	8,060,291 (16.55)	7,620,854 (16.58)	7,794,372 (15.36)	8,258,321 (17.68)	8,558,991 (17.41)	7,664,280 (17.6)

*Note*: Multiple mapped, number of reads mapped to multiple sites; uniquely mapped, number of reads mapped to one site only; Reads mapped to ‘+’ or ‘−’, mapped to ‘+’ or ‘−’ strands of DNA; Splice reads, number of reads mapped to splice sites

### Estimates of gene expression levels

To evaluate the gene expression levels, FPKM was calculated for each predicted gene. Approximately 40% of the genes (or ~5500 genes) were expressed at relatively high levels with FPKM ≥ 15; ~35% of the genes were not expressed or were expressed at low levels (FRKM = 0–3) (Table 3).

**Table 3:** The number and percentages of genes exhibiting differential expression levels as estimated using fragments per kilobase pair per million reads (FPKM)

FPKM interval	R_As24h *n* (%)	S_As24h *n* (%)	R_As13d *n* (%)	S_As13d *n* (%)	R_As19d *n* (%)	S_As19d *n* (%)
0–1	3,908 (27.23)	3,884 (27.07)	3,169 (22.08)	3,477 (24.23)	3,319 (23.13)	3,256 (22.69)
1–3	1,450 (10.10)	1,475 (10.28)	1,426 (9.94)	1,469 (10.24)	1,563 (10.89)	1,484 (10.34)
3–15	3,540 (24.67)	3,516 (24.50)	3,853 (26.85)	3,870 (26.97)	4,097 (28.55)	4,009 (27.94)
15–60	3,635 (25.33)	3,639 (25.36)	4,054 (28.25)	3,674 (25.60)	3,579 (24.94)	3,791(26.42)
> 60	1,817 (12.66)	1,836 (12.79)	1,848 (12.88)	1,860 (12.96)	1,792 (12.49)	1,810 (12.61)

### Differentially expressed gene (DEG) analysis

**Fig. 1:**
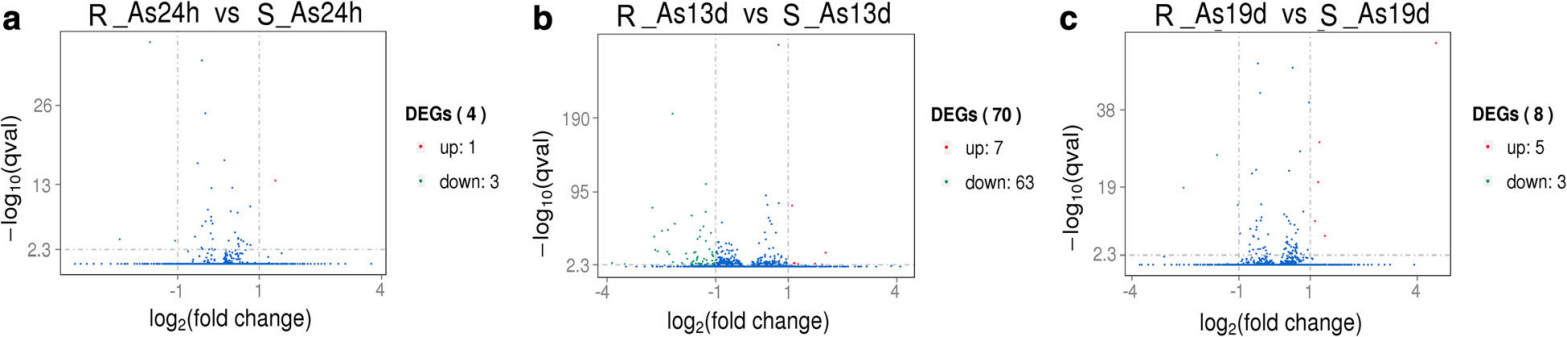
Volcano plot analysis displaying differentially expressed genes: **a** R-As24h *vs* S-As24h;**b** R-As13d *vs* S-As13d;**c** R-As19d *vs* S-As19d. The *y*-axis corresponds to the mean expression value of log10 (q-value), and the *x*-axis displays the log2 fold change value. The *red* and *green dots* represent the significantly up- and downregulated transcripts, respectively; the *blue dots* represent the transcripts whose expression levels did not reach statistical significance between the two groups

To explore the effects of infections with drug-resistant *P. yoelii* on *An. stephensi* gene transcription, we compared gene expression profiles between *An. stephensi* infected with drug-resistant *Plasmodium* (R) and *An. stephensi* infected with drug-sensitive *Plasmodium* (S) at 24 h, 13 d and 19 d after blood-feeding. The numbers of significant DEGs were 4, 70 and 8, respectively, by comparing R-As24h *vs* S-As24h, R-As13d *vs* S-As13d, and R-As19d *vs* S-As19d cDNA libraries (DEGs are presented as a Volcano plot with log_2_ (fold change) values *vs* log_10_ (q-value) values for associated experiments (Fig. 1). To validate the fold changes, we analyzed the expression patterns of nine genes randomly selected from each group by quantitative real-time PCR using the same total RNA samples, and the results showed that the experiment had good reproducibility (Additional file 1: Figure S1). These data reveal that increased variation in gene expression occurred at 13 d after blood infection upon release of *P. yoelii* sporozoites from oocysts and subsequent migration through the hemolymph. Among the 70 genes that were differentially transcribed, seven were upregulated and 63 were downregulated. To facilitate functional annotation, the observed DEGs were aligned with databases pertaining to *An. gambiae*, *Aedes aegypti*, *Culex quinquefasciatus* and other insects (e.g. *Drosophila melanogaster*) (Table 3). Nine of the observed DEGs are involved in immune responses, seven of the DEGs are involved in extracellular and intracellular signal transduction, and eight DEGs encode factors involved in oxidative stress and detoxification. Many of the DEGs are involved in cytoskeleton formation, cell adhesion and metabolic biological processes. In addition, several of the DEGS exhibited no significant sequence similarity to known proteins (Additional file 2: Table S1).

#### Pattern recognition receptors (PRRs)

Upon release of the sporozoites from oocysts, the sporozoites encounter immune responses facilitated by hemocytes and fat bodies. These responses are initiated when microbial surface molecules are recognized as ‘non-self’ by PRRs involved in binding to pathogen-associated molecular patterns. For example, in this study, we observed that genes ASTE000822 and ASTE010245 encode the peptidoglycan-recognition protein, SC2. Peptidoglycan recognition proteins (PGRPs) represent an important family of pattern recognition receptors that are involved in the activation of immune responses to parasites in mosquitoes. Presently, only PGRPLC, a peptidoglycan recognition protein isoform previously identified as a receptor of the IMD pathway in *Drosophila*, has been shown to be essential in triggering anti-*P. falciparum* responses through the IMD pathway in the gut tissue of *An. gambiae* [18]. Therefore, the exact role of PGRP-SC has not yet been fully characterized. It remains to be elucidated if SC2 plays a role during release of sporozoites from oocysts. It is possible that drug-resistant *Plasmodium* strains inhibit expression of the gene encoding for SC2 in *An. stephensi*.

#### Regulators of the JNK pathway

Dual-specificity phosphatases (DUSPs) are enzymes that remove phosphate groups from both phospho-tyrosine and phospho-serine/threonine residues. In the present study, we observed two DUSPs: ASTE005078 and ASTE007826. In *Drosophila*, Puckered has been identified as a DUSP, exhibiting specificity towards the c-Jun-N-terminal kinase (JNK) [19]. Some studies have revealed a role for Puckered in the regulation of JNK-dependent processes, including immunity and stress tolerance. The JNK pathway is a key mediator of antiplasmodial immunity in *An. gambiae* [20], and suppression of this pathway greatly enhanced *Plasmodium* infection [21]; over-activation of this cascade, following silencing of the suppressor, Puckered, resulted in the opposite effect. Thus, it is possible that in drug-resistant *P. yoelii*, Puckered induced over-activation of the JNK pathway.

#### Phagosome pathway

Actin is a highly versatile, abundant, and conserved protein that functions in a variety of intracellular processes [22]. In this study, we observed two genes that encode actin: ASTE005787 and ASTE005788. These proteins were categorized into the phagosome pathway following KEGG analysis. Recent studies have revealed that insect cytoplasmic actin is an extracellular pathogen recognition factor that mediates antibacterial defense mechanisms. Furthermore, insect actin proteins are secreted from cells upon immune challenge through an exosome-independent pathway [22]. Moreover, actin from *An. gambiae* interacts with the extracellular MD2-like immune factor, AgMDL1, which binds to the surfaces of bacteria and mediates their phagocytosis. Thus, mosquito actin represents a *Plasmodium* infection antagonist [22].

#### Regulator of the melanization response

Recognition of non-self in insects may trigger modulatory proteolytic cascades of serine proteases that, depending on the strength of the recognition signal, can activate downstream immune effector reactions, including antimicrobial peptide (AMP) synthesis, hemolymph clotting or melanization [23]. In the present study, we observed one easter-like serine protease, ASTE009034. Serine proteases are a class of enzymes characterized by the presence of an invariant catalytic triad consisting of His, Asp and Ser. These enzymes play important roles in the regulation of immune responses of invertebrates by proteolytically cleaving and activating downstream members of these pathways [24]. In some invertebrates, serine proteases are involved in the activation of the enzyme prophenoloxidase, resulting in the targeted deposition of melanotic capsules around invasive organisms [25]. Serine proteases are a class of enzymes characterized by the presence of an invariant catalytic triad consisting of His, Asp and Ser. These enzymes play important roles in the regulation of immune and coagulation responses of both vertebrates and invertebrates, Serine proteases are a class of enzymes characterized by the presence of an invariant catalytic triad consisting of His, Asp and Ser. These enzymes play important roles in the regulation of immune and coagulation responses of both vertebrates and invertebrates.

#### Activator of complement reaction

Leucine-rich repeat-containing proteins are pivotal to host defense responses in both plants and animals [26]. In this study, we observed one leucine-rich repeat-containing protein, ASTE010296. In *An. gambiae*, two such proteins, LRIM1 and APL1C, antagonize malaria infections; these proteins circulate in the hemolymph as high-molecular-weight complexes held together by disulfide bridges [26]. These complexes interact with the complement C3-like protein, TEP1, promoting its cleavage or stabilization and its subsequent localization on the surface of midgut-invading *P. berghei* parasites, thereby targeting their destruction.

#### Insulin signaling cascade

The insulin signaling cascade plays a central role in regulating immune and oxidative stress responses. This cascade affects the life span of the fruit fly *D. melanogaster* [27]. In this study, we observed one insulin-like growth factor-binding protein, ASTE005729. In mosquitoes, insulin signaling regulates key steps in egg maturation and immunity, and the latter may critically influence the capacity of mosquitoes to effectively transmit malaria parasites. Studies have demonstrated that molecules from both the invading parasite and associated blood meal elicit functional responses in female mosquitoes. These responses are regulated through the insulin signaling pathway or cross-talk with interacting pathways.

#### Oxidative stress and detoxification proteins

Several genes for oxidative stress and detoxification were differentially expressed following infection with drug-resistant parasite. Some of these genes were observed to be involved in the downregulation of peroxidase and cytochrome P450 and the upregulation of peroxiredoxin. Previous studies identified a heme peroxidase (HPX2) and NADPH oxidase 5 (NOX5) as critical mediators of midgut epithelial nitration and antiplasmodial immunity that can enhance nitric oxide toxicity in *An. gambiae*. These studies also revealed that target ookinete-epithelial nitration works sequentially with thioester-containing protein 1 (TEP1)-mediated lysis [28]. Thus, it is possible that the peroxidase exhibits antiplasmodial immunity during sporozoite release (from oocysts) and migration through the hemolymph. In addition, peroxiredoxins (Prxs) are enzymes that are known to detoxify reactive oxygen species (ROS) and reactive nitrogen oxide species (RNOS) [29]. It is likely that infections caused by drug-resistant *P. yoelii* induce the upregulation of peroxiredoxins and the subsequent detoxification of ROS and RNOS.

#### Iron metabolic biological processes

In this study, we observed that the drug-resistant NSM parasite can downregulate some genes (ASTE001964, ASTE011592 and ASTE001957) involved in iron metabolism in *Anopheles*. Iron participates in many vital processes in insects, including the vitellogenic process. In *An. gambiae* laboratory strains, refractoriness to *Plasmodium* infection is associated with an increased capacity to generate ROS, which tightly linked to Fe and Mn metabolism [30]. Further understanding iron transport in *Anopheles* may provide physiological clues to design alternative strategies for vector control aimed at interrupting drug-resistant *Plasmodium* development by regulating iron availability.

### GO enrichment analysis of DEGs

**Fig. 2:**
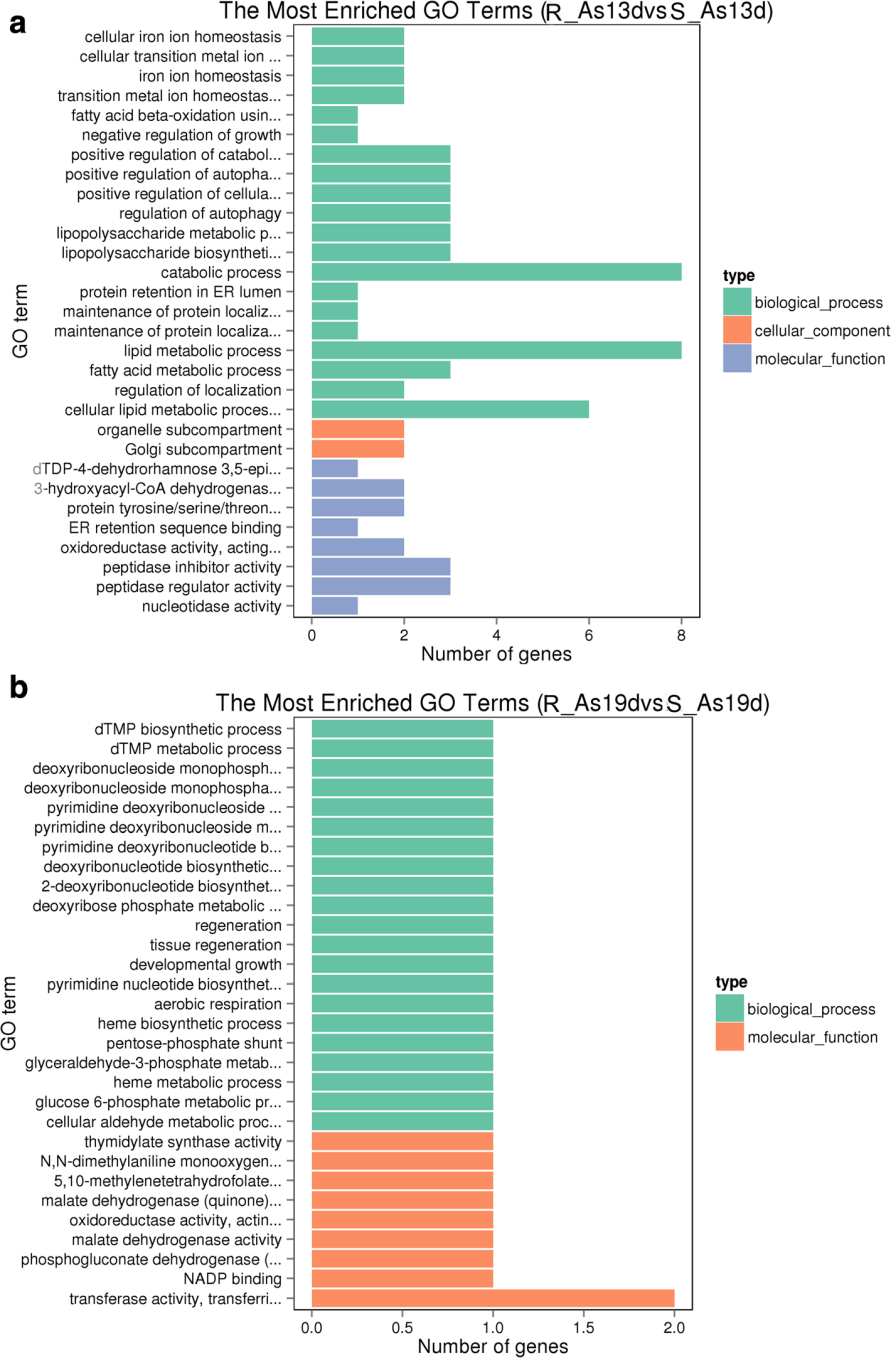
Bar graph of Gene Ontology (GO) enrichment analysis. Enrichment of gene ontology terms in differentially expressed sequences for: **a** R-As13d *vs* S-As13d; **b** R-As19d *vs* S-As19d

To further investigate the functional associations of differentially expressed genes, we performed gene ontology (GO) analysis using the GO seqR package [31]. The majority of the DEGs were significantly represented in the three main GO categories: ‘biological process’,‘molecular function’and‘cell component’. GO terms with corrected *P*-values < 0.05 were considered significantly enriched by differentially expressed genes. Cellular iron ion homeostasis, regulation of autophagy, lipopolysaccharide metabolic processes, peptidoglycan catabolic process, protein tyrosine/serine/threonine, peptidase inhibitors and regulator activity were enriched in the R-As13d library compared with the S-As13d library; dTMP metabolic processes and transferase activity were highly enriched in R-As19d *vs* S-As19d (Fig. 2, Additional file 3: Table S2).

### Functional classification by KEGG

KEGG is a database resource for understanding high-level functions and utilities of the biological system, including the cell, the organism and the ecosystem [32]. Pathway-based analysis facilitates an understanding of the biological functions of gene products [33]. Using this classification system, we individually observed 1,18 and 4 pathways in R-As24h *vs* S-As24h, R-As13d *vs* S-As13d, and R-As19d *vs* S-As19d cDNA libraries, respectively (Additional file 4: Table S3). We were interested in functions that correlated with phagosome and phenylalanine metabolism pathways. Recent studies have suggested that phagocytosis and encapsulation or melanization cascades play important roles in *Plasmodium* during sporozoite release (from oocysts) and migration through the hemolymph [34, 35].

Through comprehensive analysis of DEGs, GO enrichment and functional classification by KEGG, we can conclude that the variation of gene expression in *An. stephensi* infected with the drug-resistant *P. yoelii* NSM occurred mainly 13 days after blood meal during sporozoite migration through the hemolymph and that the DEGs may involve some important immune reaction and iron metabolism pathways. The gene variations may influence and generate some selective pressures for the development of drug-resistant *P. yoelii* NSM in the mosquito, which can give rise to drug-resistant polymorphisms in the parasite. However, potential drug-resistant polymorphisms generated in mosquitoes are rarely considered, despite the fact that stage of insect represents the life-cycle stage where the majority of parasite genetic recombination events occur.

Several studies have shown that *Plasmodium* can harbor highly host-specific drug-resistant polymorphisms, most likely reflecting different selective pressures found in humans and mosquitoes [36, 37]. In addition, field studies have reported that vectors might impose specific selection pressures on pathogens. Indeed, a molecular characterization of *Borrelia burgdorferi* (*sensu lato*) performed in Slovenia revealed significant differences between tick and human isolates [38]. A separate study revealed that selective pressures exerted by the *Aedes* mosquito play important roles in the generation of polymorphisms in Dengue virus [39]. Furthermore, contrasting antifolate resistance polymorphisms were observed in *P. falciparum* when parasites isolated from human blood were compared with parasites isolated from *Anopheles arabiensis* found in sleeping huts in rural Zambia [37]. With the further determination of drug-resistant genes in the *P. yoelii* NSM, we can further analyze the gene polymorphisms in the mosquito. We also observed the basic biological profile of the drug-resistant *P. yoelii* NSM in *An. stephensi*; however, there were no significant variations regarding oocyst and sporozoite numbers between mosquitoes infected with the drug-resistant NSM and the drug-sensitive parasites. Our preliminary analysis of this result is that it reflects a comprehensive effect of the variations of biological features and the immune reaction of the mosquito. However, we also recognize that some polymorphisms unrelated to drug resistance may exist between NSM and NS generated during the drug selection process and that these unknown differences may also contribute to the observed variation in gene expression.

## Conclusion

Our study shows that drug-resistant *P. yoelii* NSM have an impact on the transcript abundance levels of *An. stephensi* occurring mainly 13 days after blood meal during sporozoite migration through the hemolymph, leading to downregulation of many genes in immune responses, such as the phagosome pathway, melanization and complement activation. In addition, some genes implicated in iron metabolism pathways in *An. stephensi* were also downregulated. However, it remains to be elucidated whether these DEGs can become effective selective pressures for drug-resistant polymorphisms of *Plasmodium* in the mosquito and whether these gene variations can lead to transmission of drug-resistant *P. yoelii* by *An. stephensi*.

## Additional files

Additional file 1: Figure S1. Confirmation of the RNA sequencing expression profiling by qRT-PCR using the same RNA samples. Nine differentially expressed genes were selected for validationof the expression data. (DOC 64 kb)

Additional file 2: Table S1. Differential expression analysisof *An. stephensi* induced by drug-resistant *Plasmodium* at the three major spatial transition stages of *Plasmodium* infections. (DOCX 25 kb)

Additional file 3: Table S2. Gene Ontology (GO) enrichment analysis of differentially expressed genes: (A) R-As13d *vs* S-As13d; (B) R-As19d *vs* S-As19d. (XLS 249 kb)

Additional file 4: Table S3. KEGG analysis of differentially expressed genes: (A) R-As24h *vs* S-As24h; (B) R-As13d *vs* S-As13d; (C) R-As19d *vs* S-As19d. (XLS 34 kb)

## Abbreviations

AMPs: Antimicrobial peptides
cDNA: Complementary DNA
DEGs: Differentially expressed genes
DUSPs: Dual-specificity phosphatases
FPKM: Fragments per kilobase per millions
GO: Gene ontology
iRBCs: Infected red blood cells
JNK: C-Jun-N-terminal kinase
NSM: *Plasmodium. y. nigeriensis* NSM
PCR: polymerase chain reaction
PGRPs: Peptidoglycan recognition proteins
PRRs: Pattern recognition receptors
rpS7: Ribosomal protein S7
