# Supplementary material for: Differential gene expression in Anopheles stephensi following infection with drug-resistant Plasmodium yoelii
Source: Parasit Vectors. 2017 Aug 29;10:401. doi: 10.1186/s13071-017-2326-y (PMC5576267; doi:10.1186/s13071-017-2326-y)
Supplement: Supplementary file 1 — Confirmation of the RNA sequencing expression profiling by qRT-PCR using the same RNA samples. Nine differentially expressed genes were selected for validationof the expression data. (DOC 64 kb) [file 13071_2017_2326_MOESM1_ESM.doc]

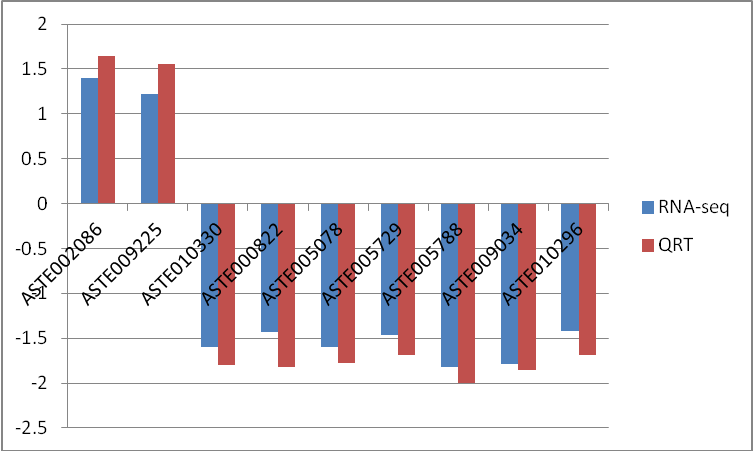


**Figure S1.** Confirmation of the RNA-Seq expression profiling by qRT-PCR using the same RNA samples (R-As24h, S-As24h, R-As13d, S-As13d, R-As19d, and S-As19d). Nine differential expressed genes were selected for validation of the expression data. These genes are ASTE002086，ASTE009225，ASTE010330，ASTE000822，ASTE005078，ASTE005729，ASTE005788，ASTE009034，ASTE010296. The ribosomal protein S7 (rpS7, AF539918) mRNA was used for normalization. The primer pair and amplicon size are: 5'- GTGTTTGTTG

CACTGCTGCT -3’, 5'- GTGGAATGCTTCGCTTTAGG -3' and 118 bp for ASTE002086, 5'- AACTCGGACGTGCATTTCTC -3', 5'- ACAGGTCCTTGTCGTTGGAC -3' and 112 bp for ASTE009225, 5'- CCGGTAAAGTTCTCGGACAC -3', 5'- TAGGGTTGGTGTGGTCCAAT -3' and 120 bp for ASTE010330, 5'- ACATCGCCTCCAACTACTGG -3', 5'- CTACACGTTCGG

GTTGAAGC -3’ and 116bp for ASTE000822, 5'- TTTACGGTTGATCGGTGTCA -3', 5'- ATCGGGAAGCCCATATACCT -3' and 123 bp for ASTE005078, 5'- TGACAATGCTCGA

TCTCTCG -3', 5'- AACTCGATGTGGGAGATGCT -3' and118 bp for ASTE005729, 5'- GCT

AACCGCGAGAAGATGAC -3', 5'- ACCCGAGTCCAGCACAATAC -3' and 129 bp for ASTE005788, 5'- CAGCATCAATCGCGTCTTTA -3', 5'- AAGAGTTCCGCCACAGTGAT -3' and 120 bp for ASTE009034, and 5’- GCTCGAACTCAACCAGAACC -3’, 5’-TGGAAC

ACGCATAGATCGAG -3’ and117 bp for ASTE010296, 5’-CTAACGACACGAAGACCACA

AGA-3’, 5’-CAACCTGCAACGAAGCAAAA-3’ and 81 bp for rpS7Annealing temperatures of the qRT-PCRs for all transcripts are 60ºC.
