# Supplementary material for: Differential gene expression in Anopheles stephensi following infection with drug-resistant Plasmodium yoelii
Source: Parasit Vectors. 2017 Aug 29;10:401. doi: 10.1186/s13071-017-2326-y (PMC5576267; doi:10.1186/s13071-017-2326-y)
Supplement: Supplementary file 2 — Differential expression analysisof An. stephensi induced by drug-resistant Plasmodium at the three major spatial transition stages of Plasmodium infections. (DOCX 25 kb) [file 13071_2017_2326_MOESM2_ESM.docx]

Table S1: The differential expression analysis of *An. stephensi* induced by drug-resistant *Plasmodium* at the three major spatial transition stages of *Plasmodium* infections

| Groups | Gene_id | log2.Fold_change. | Blast swiss prot | |  |  |  |
| --- | --- | --- | --- | --- | --- | --- | --- |
| R-As24h vs S-As24h | **metabolic biological processes** | |  |  |  |  |  |
|  | ASTE010015 | -1.0667 | sp\|P43166\|Carbonic anhydrase | | |  |  |
|  | unkown function | |  |  |  |  |  |
|  | ASTE002086 | 1.398 | -//- |  |  |  |  |
|  | ASTE011497 | -1.6801 | -//- |  |  |  |  |
|  | ASTE011498 | -2.4228 | -//- |  |  |  |  |
| R-As13d *vs* S-As13d | **immune reaction** | |  |  |  |  |  |
|  | ASTE000822 | -1.4369 | sp\|Q70PU1\|Peptidoglycan-recognition protein SC2 | | | |  |
|  | ASTE005078 | -1.598 | sp\|Q9UII6\|Dual specificity protein phosphatase 13 isoform B | | | | |
|  | ASTE005729 | -1.4621 | sp\|P35859\|Insulin-like growth factor-binding protein complex acid labile subunit | | | | |
|  | ASTE005787 | -1.5007 | sp\|P49128\|Actin-1 | |  |  |  |
|  | ASTE005788 | -1.8199 | sp\|P49128 Actin-1 | |  |  |  |
|  | ASTE007826 | -2.1847 | sp\|O95147\|Dual specificity protein phosphatase 14 | | | |  |
|  | ASTE009034 | -1.7854 | sp\|P13582\|Serine protease easter | | |  |  |
|  | ASTE010245 | -1.2187 | sp\|Q9VS97\| Peptidoglycan-recognition protein SD | | | |  |
|  | ASTE010296 | -1.4219 | sp\|Q2I0M4\| Leucine-rich repeat-containing protein 26 | | | | |
|  | **Extracellular and intracellular signal transduction** | | | |  |  |  |
|  | ASTE001413 | -1.0356 | sp\|P08217\| Chymotrypsin-like elastase family | | | |  |
|  | ASTE002262 | -1.5139 | sp\|Q8R422\| CD109 antigen | | |  |  |
|  | ASTE002667 | -1.2772 | sp\|P34455\| Probable aconitate hydratase | | | |  |
|  | ASTE004507 | -1.0063 | sp\|Q29513\| Glycine N-methyltransferase | | | |  |
|  | ASTE005726 | -1.1883 | sp\|P48728\| Aminomethyltransferase | | |  |  |
|  | ASTE008182 | -1.4705 | sp\|Q6YHK3\| CD109 antigen | | |  |  |
|  | **Oxidative stress and detoxification** | | |  |  |  |  |
|  | ASTE002173 | -1.2699 | sp\|Q62425\| Cytochrome c oxidase subunit | | | |  |
|  | ASTE002872 | -2.6678 | sp\|Q9VEG6\| Chorion peroxidase | | |  |  |
|  | ASTE004682 | 2.0329 | sp\|Q5ZJF4\| Peroxiredoxin-6 | | |  |  |
|  | ASTE006656 | -1.0952 | sp\|Q8MP06\| Senecionine N-oxygenase | | |  |  |
|  | ASTE007944 | -1.0679 | sp\|P19967\| Cytochrome b5-related protein | | | |  |
|  | ASTE008792 | -2.4846 | sp\|Q9JHI8\| NADPH oxidase 4 | | |  |  |
|  | **Cytoskeleton and cell adhesion** | | |  |  |  |  |
|  | ASTE002132 | -1.1871 | sp\|Q7M4F3\| Endocuticle structural glycoprotein SgAbd-2 | | | | |
|  | ASTE002335 | -1.5012 | sp\|Q9I7U4\|Titin | |  |  |  |
|  | ASTE002886 | -1.385 | sp\|Q9VEN1\| Filamin-A | |  |  |  |
|  | ASTE004453 | -1.3598 | sp\|P35554\| Flightin | |  |  |  |
|  | ASTE005320 | -1.6414 | sp\|O01761\|Muscle M-line assembly protein | | | |  |
|  | ASTE005624 | -1.6613 | sp\|A1ZA47\|PDZ and LIM domain protein Zasp | | | |  |
|  | ASTE006206 | -2.3327 | sp\|P47949\|TNNC3_DROME Troponin C, isoform 3 OS=Drosophila melanogaster GN=TpnC73F PE=2 SV=2//1.38373e-23 | | | | |
|  | ASTE010079 | -1.5495 | sp\|Q3UIZ8\| Myosin light chain kinase | | |  |  |
|  | ASTE010640 | -1.2713 | sp\|Q23551\| Twitchin | |  |  |  |
|  | ASTE011453 | -2.7472 | sp\|A1ZA47\| PDZ and LIM domain protein Zasp | | | |  |
|  | sensory receptors | |  |  |  |  |  |
|  | ASTE010148 | 1.9342 | sp\|P54193\|General odorant-binding protein 83a | | | |  |
|  | ASTE010078 | 1.1096 | sp\|P48994\| Transient-receptor-potential-like protein | | | |  |
|  | **metabolic biological processes** | |  |  |  |  |  |
|  | ASTE001964 | -1.0937 | sp\|Q9D7P6\|Iron-sulfur cluster assembly enzyme ISCU | | | |  |
|  | ASTE003745 | 1.739 | sp\|Q16927\| Vitellogenin-A1 | | |  |  |
|  | ASTE004175 | -1.4353 | sp\|Q6GQV7\| differentiation-related factor | | | |  |
|  | ASTE004490 | -1.6537 | sp\|W4VS53\| CRISP | |  |  |  |
|  | ASTE004901 | -1.5228 | sp\|Q92820\| Gamma-glutamyl hydrolase | | |  |  |
|  | ASTE005159 | -1.0758 | sp\|A5PK46\|Pancreatic lipase-related protein | | | |  |
|  | ASTE006082 | 1.275 | sp\|O76217\| Peritrophin-1 | |  |  |  |
|  | ASTE006408 | -1.8753 | sp\|O46040\|Protein msta, isoform A | | |  |  |
|  | ASTE007267 | -1.4279 | sp\|Q9VFS2\| Carotenoid isomerooxygenase | | | |  |
|  | ASTE007781 | -1.0063 | sp\|Q91XV4\| L-xylulose reductase | | |  |  |
|  | ASTE008207 | 1.1543 | sp\|P40807\|Ornithine decarboxylase 1 | | |  |  |
|  | ASTE008637 | -2.2886 | sp\|P00819\| Acylphosphatase-2 | | |  |  |
|  | ASTE008757 | -1.0233 | sp\|Q96RW7\| Hemicentin-1 | |  |  |  |
|  | ASTE010013 | -1.0415 | sp\|Q7PQV7\| ADP,ATP carrier protein | | |  |  |
|  | ASTE010995 | -1.2547 | sp\|Q5SRE7\| Phytanoyl-CoA dioxygenase domain-containing protein 1 | | | | |
|  | ASTE011526 | -1.4922 | sp\|Q9VSY6\|Phosphoserine phosphatase | | | |  |
|  | ASTE011592 | -2.1303 | sp\|Q26643\| Transferrin | |  |  |  |
|  | ASTE011715 | -1.0756 | sp\|Q3SXM5\|Inactive hydroxysteroid dehydrogenase-like protein 1 | | | | |
|  | **unkown function** | |  |  |  |  |  |
|  | ASTE000162 | 1.181 | -//- |  |  |  |  |
|  | ASTE000561 | -2.6992 | -//- |  |  |  |  |
|  | ASTE000590 | -1.9291 | -//- |  |  |  |  |
|  | ASTE001957 | -2.3206 | -//- |  |  |  |  |
|  | ASTE001958 | -1.2328 | -//- |  |  |  |  |
|  | ASTE002145 | -2.6021 | -//- |  |  |  |  |
|  | ASTE004106 | -2.1911 | -//- |  |  |  |  |
|  | ASTE005235 | -1.5557 | -//- |  |  |  |  |
|  | ASTE006008 | -1.2718 | -//- |  |  |  |  |
|  | ASTE007003 | -1.3404 | -//- |  |  |  |  |
|  | ASTE007048 | -1.3196 | -//- |  |  |  |  |
|  | ASTE007979 | -1.6864 | -//- |  |  |  |  |
|  | ASTE008996 | -1.131 | -//- |  |  |  |  |
|  | ASTE009535 | -1.0763 | -//- |  |  |  |  |
|  | ASTE010100 | -1.1722 | -//- |  |  |  |  |
|  | ASTE011497 | -1.6166 | -//- |  |  |  |  |
|  | ASTE014216 | -3.5125 | -//- |  |  |  |  |
|  | ASTE014673 | -3.8625 | -//- |  |  |  |  |
| R-As19d *vs* S-As19d | **Oxidative stress and detoxification** | | |  |  |  |  |
|  | ASTE004682 | -2.5537 | sp\|Q5ZJF4\|Peroxiredoxin-6 | | |  |  |
|  | ASTE006656 | 1.2579 | sp\|Q8MP06\|Senecionine N-oxygenase | | |  |  |
|  | **Extracellular and intracellular signal transduction** | | | |  |  |  |
|  | ASTE010330 | -1.6113 | sp\|Q27289\| Chymotrypsin-1 | | |  |  |
|  | **metabolic biological processes** | |  |  |  |  |  |
|  | ASTE001658 | -1.0319 | sp\|P84239\|Histone H3 | |  |  |  |
|  | ASTE009225 | 1.2223 | sp\|P50887\|60S ribosomal protein L22 | | |  |  |
|  | ASTE010779 | 1.4125 | sp\|Q6GNL7\| Cytosolic 10-formyltetrahydrofolate dehydrogenase | | | | |
|  | **unkown function** | |  |  |  |  |  |
|  | ASTE011497 | 4.5247 | -//- |  |  |  |  |
|  | ASTE011500 | 1.1331 | -//- |  |  |  |  |
